# Analysis of echolocation behavior of bats in “echo space” using acoustic simulation

**DOI:** 10.1186/s12915-022-01253-y

**Published:** 2022-03-14

**Authors:** Yu Teshima, Yasufumi Yamada, Takao Tsuchiya, Olga Heim, Shizuko Hiryu

**Affiliations:** 1 Faculty of Life and Medical Sciences, Doshisha University, Kyōtanabe, Kyoto Japan; 2 Department of Mathematical and Life Sciences, Hiroshima University, Higashihiroshima, Hiroshima Japan; 3 Faculty of Sciences and Engineering, Doshisha University, Kyōtanabe, Kyoto Japan

**Keywords:** Bats, Acoustic simulation, Flight navigation, Active sensing

## Abstract

**Background:**

Echolocating bats use echo information to perceive space, control their behavior, and adjust flight navigation strategies in various environments. However, the echolocation behavior of bats, including echo information, has not been thoroughly investigated as it is technically difficult to measure all the echoes that reach the bats during flight, even with the conventional telemetry microphones currently in use. Therefore, we attempted to reproduce the echoes received at the location of bats during flight by combining acoustic simulation and behavioral experiments with acoustic measurements. By using acoustic simulation, echoes can be reproduced as temporal waveforms (including diffracted waves and multiple reflections), and detailed echo analysis is possible even in complex obstacle environments.

**Results:**

We visualized the spatiotemporal changes in the echo incidence points detected by bats during flight, which enabled us to investigate the “echo space” revealed through echolocation for the first time. We then hypothesized that by observing the differences in the “echo space” before and after spatial learning, the bats’ attentional position would change. To test this hypothesis, we examined how the distribution of visualized echoes concentrated at the obstacle edges after the bats became more familiar with their environment. The echo incidence points appeared near the edge even when the pulse direction was not toward the edge. Furthermore, it was found that the echo direction correlated with the turn rate of the bat’s flight path, revealing for the first time the relationship between the echo direction and the bat’s flight path.

**Conclusions:**

We were able to clarify for the first time how echoes space affects echolocation behavior in bats by combining acoustic simulations and behavioral experiments.

**Supplementary Information:**

The online version contains supplementary material available at 10.1186/s12915-022-01253-y.

## Background

Humans rely heavily on vision as a result of their exceptionally high visual acuity [1]. This superiority of human vision has guided the direction of technological development in recent years. For instance, image recognition and simultaneous localization and mapping technologies using stereo and monocular cameras, such as automated driving, have advanced rapidly, particularly in combination with deep learning [2, 3]. The accuracy of image processing technology has significantly improved in recent years and hence, is an important technological element in the field of sensing engineering. In contrast to humans, other animals optimize their behavior in weak vision or grasp their environment with sensory organs other than vision. For instance, echolocating bats mainly operate in the dark (e.g., caves and at night) where they cannot rely on vision but can perceive their environment via acoustic sensing [4]. They actively ensonify their environment to collect information contained in returning echoes to navigate safely and to find and track prey successfully.

Previous research showed that bats adapt their sonar and flight behavior depending on environmental characteristics [5–8]. In this and other studies, sonar behavior is represented by acoustic characteristics of pulses and pulse direction [9, 10]. However, information on echoes is often missing. Since bats use echoes to perceive space and control their behavior or plan their flight path, it is necessary to analyze the echoes they receive in order to understand their behavior. However, it is also difficult to measure all the echoes reaching both ears of a bat during flight, even with a conventional telemetry microphone [11] or an onboard acoustic logger [12]. Kothari et al. [13] uses a simple echo model to obtain 3D locations of echo incidence points based on geometric instantenous distances between bats and objects (cylinders). By using a cylinder as a target, the timing of receiving the echoes by the bat can be determined as single sound source point based on the physics of acoustics (Additional file 1: Movie S1). However, in the natural environment, it is unlikely that each of the individual objects that make up a complex spatial structure can be assumed to be a single source (Additional file 2: Movie S2). Therefore, in order to derive the echo space, it is necessary to develop a method to acquire the echoes received by both ears based on the propagation phenomenon of sound wave in obstacle space.

In this study, we developed a method to reproduce the echoes reaching the right and left ears of bats in flight using flight and pulse information obtained from behavioral experiments into the acoustic simulation space. Using this new method, we aimed at understanding the sonar and flight behavior of bats from a previous behavioral study [14]. In that study, bats were allowed to fly repeatedly through an obstacle course while their sonar activity and flight path were recorded. In the first step, we aimed at understanding how the information from echoes (“echo space”) changes with an increased experience of the bats across a series of flights.

We hypothesized that along with previously measured reductions in pulse emission number and gaze angle, the locations of echo incidence points should also change enabling bats to optimize their flight path. We expected to find a more focused pattern of echo incidence points during the last flight compared to the first, since bats should receive sufficient information to evade the obstacles while emitting a smaller number of pulses at the same time. In the second step, we aimed at investigating the influence of echo incidence points on the more immediate flight behavior in the context of flight path planning. In a previous study [15], it was shown by correlation that the pulse information precedes the turn rate and concluded that the pulse direction of bats behaves in the same way as the gaze of a car driver [16–18]. However, in contrast to vision, pulse direction is not equivalent to the echo direction (the angular difference between the echo incident point and the bat’s flight direction) and therefore needs to be considered separately. We hypothesized that given the principle of echolocation, the information conveyed by the echoes should have a similar or more profoundly important relationship with the pulse direction and turn rate. Therefore, we investigated the correlation between the direction of the echo and the turn rate. Furthermore, we also expected to find an influence of the learning process on the results of this comparision, as bats might use spatial memory during the last flight to a stronger degree compared to the first flight. After testing these hypotheses, the “echo space” changed before and after the bats learned the space, and the echo incidence points were concentrated at the edges of the obstacles. We also found that the echo direction and the turn rate were correlated, as were the pulse direction and the turn rate, and that the spatial learning effect shifts the location of the peak of the correlation coefficient. These results present the importance of including echoes to examine the echolocation behavior of bats.

## Results

In a behavioral experiment, bats (*Rhinolophus ferrumequinum nippon*) were allowed to fly repeatedly through an obstacle course made up by acrylic plates, and their flight trajectories and pulse information (direction, intensity, and timing of emission) were measured [14]. The same obstacle course layout was constructed as a two-dimensional acoustic simulation space. With the information on the positions and pulses of the bats acquired in the behavioral experiment, we could determine the echoes that reached the positions of the right and left ears of the bats in flight in the simulation using the finite-difference time-domain (FDTD) method. Based on the left and right echo delay times we then estimated the echo incidence points and visualized them as the echo space (Additional file 3: Movie S3). We chose a 2D space instead of a 3D space to reduce the processing time and memory usage of the FDTD calculations. In addition, the bats avoided obstacles and maintained an overall horizontal path in this experimental setup. Therefore, the height information is not essential (for more details, see the “Methods” section).

### Spatial learning effects on echo incidence point distribution

**Fig. 1:**
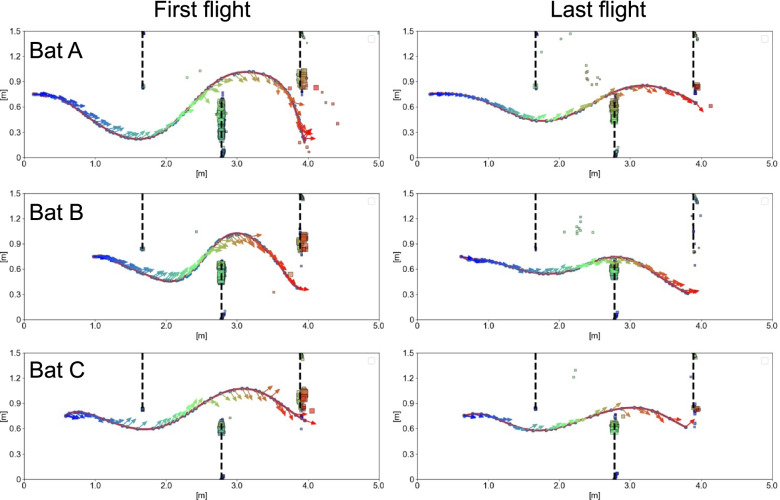
Flight direction, pulse directions, and echo incidence points during the first and last flight. Subplots of exemplary flight paths depict the flight directions (red solid lines), pulse directions (arrows), and echo incidence points (square plots) of individual bats (Bat A, Bat B, and Bat C) during the first flight and last flight. The color of the echo incidence points is the same as the color of the pulse from which the echo incidence point was generated, and its size is proportional to the sound pressure of the echo. Please note that one emitted pulse can result in several echo incidence points

**Fig. 2:**
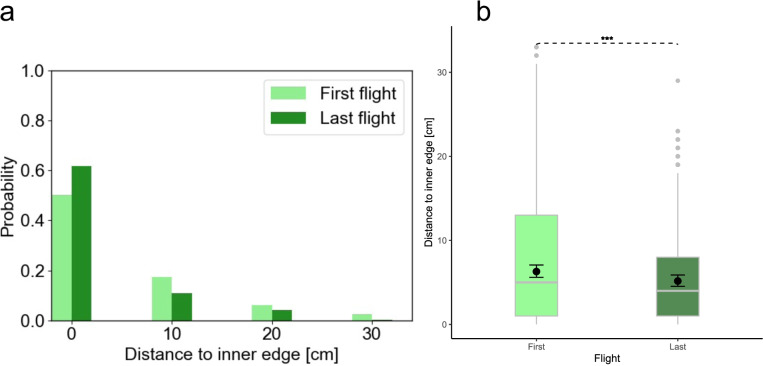
Distribution of echo incidence points on obstacle walls during the first and last flight. **a** The histogram shows the probability of echo incidence points being located at various distances to the inner edge of the obstacle (acrylic walls) during the first and last flight. **b** Box plots summarize data on the distances between echo incidence points and the inner edge within the inner half of obstacle walls for the first and last flight, while the black whisher plots represent model-related means (black circle) and associated 95% confidence intervals. These results are based on a model that fit the data well (based on residual plots) and that explained significantly more variance than its null model (*χ*^2^ = 12.95, df = 1, *p* < 0.001). In addition, the effect of the spatial-learning-factor in the model was found to be significant (*χ*^2^_type-II-Wald_ = 13.0, df = 1, *p* < 0.001)

The echo incidence points were mostly concentrated near the inner edge of the obstacle wall (Figs. 1 and 2a; see data repository for data on the other four individuals). Therefore, we selected those echo incidence points that were located within the inner half of the obstacle wall to investigate the effect of spatial learning on their distribution. We modeled the distance from the echo incidence points to the inner edge of the walls using generalized linear mixed effect models. We found that echo incidence points after spatial learning were located closer to the inner edge than before spatial learning (contrast_First/Last_: ratio = 1.2 ± 0.07 SE, df = 1053, *t*-ratio = 3.6, *p* < 0.001, Fig. 2b). The average distance of echo incidence points to the inner wall edge during the first flight was at 6.3 ± 0.4 SE cm, while the average distance was 5.2 ± 0.3 SE cm during the last flight.

### Flight path planning and spatial learning

**Fig. 3:**
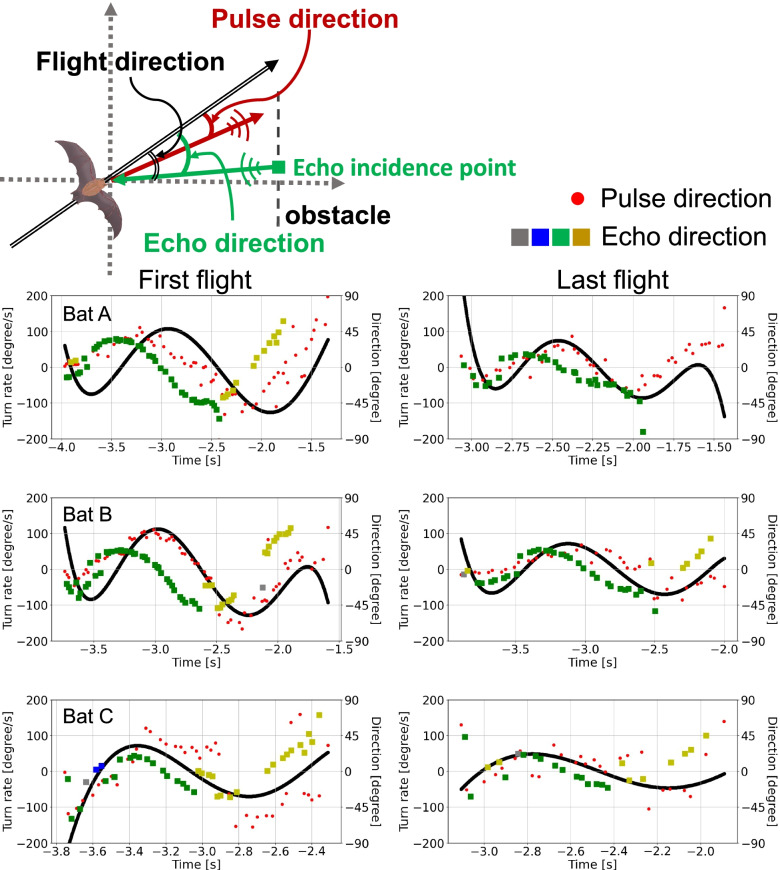
Time-series plots (first flight and last flight): pulse direction and turn rate, echo direction and turn rate. Time-series plot of the pulse direction (red rhombus plot), the echo direction (square plot), and turn rate (black solid line) in the first flight and last flight for each bat. The echo direction plot shows the echo incidence points with the highest sound pressure generated by a single pulse. The color of the echo direction plot depends on the obstacle (acrylic plate) on which the echo incidence is localized: blue indicates the first acrylic plate, green the second acrylic plate, yellow the third acrylic plate, and gray the echo incidence point that was not localized on the obstacle

**Fig. 4:**
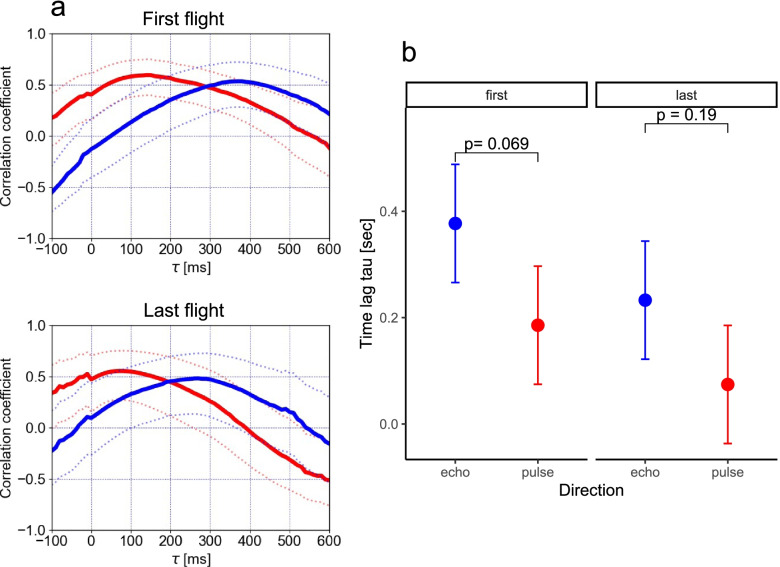
Correlation coefficients: pulse and echo direction and respective turn rate for first and last flight. **a** Correlation coefficients between the pulse direction and turn rate (solid red line) and between the echo direction and turn rate (solid blue line). The dotted lines represent 95% confidence intervals. **b** The statistical comparison of individual-specific time lag data shows means (circles) and 95% confidence intervals (whiskers) for echo and pulse directions during the first and last flight. These results are based on a model that fit the data well (based on residual plots) and that explained significantly more variance than its null model (parametric bootstrap test, stat = 16.0, df = 3, *p* = 0.007). The effect of the spatial-learning-factor in interaction with the type of information (echo or pulse) was found to be not significant (*χ*^2^_type-II-Wald_ = 0.12, df = 1, *p* = 0.7) while the single effects showed a clear effect (flight (first vs. last): χ^2^_type-II-Wald_ = 7.07, df = 1, *p* = 0.008; information (echo vs. pulse): *χ*^2^_type-II-Wald_ = 13.25, df = 1, *p* < 0.001)

It has been suggested in a previous study that the direction of pulse emission precedes the turn rate change in a bat’s flight toward a target [15]. To investigate the relationship between the pulse direction and the turn rate, and echo direction and the turn rate, we plotted individual-specific time-series (Fig. 3; see data repository for data on all the individuals) and found that although both, pulse and echo direction changes preceded the turn rate change, the echo direction changed more smoothly and preceded the turn rate change with a larger time lag than the pulse direction (Fig. 4a). To test this observation statistically and any corresponding effects of spatial learning, we determined the individual-specific time delay *τ*_max_ at which a pulse or an echo direction, respectively, are maximally correlated to a turn rate. After modeling *τ*_max_ as a function of the bat’s experience in interaction with the type of direction (echo or pulse), we found that, during the first flight, pulse and echo direction changes preceded the turn rate change at 186 ± 54 and 377 ± 54 ms, respectively. In this case, the echo direction change tended to precede the turn rate with a larger time lag than that of the pulse direction change (contrast_Echo/Pulse_: ratio = 191 ± 68 SE, df = 18, *t*-ratio = 2.8, *p* = 0.069, Fig. 4b). During the last flight, the time lags between the pulse and echo direction changed and the turn rate change decreased slightly to 74 ± 54 and 233 ± 54 ms, respectively (Fig. 4b). Also, the difference between time lags of echo and pulse direction changes decreased slightly (contrast_Echo/Pulse_: ratio = 159 ± 68 SE, df = 18, *t*-ratio = 2.3, *p* = 0.19, Fig. 4B). Based on the inter-peak time lag difference between echo and pulse direction of 191 ms (= 377–186 ms) during the first flight, we estimated that an echo direction might affect the direction of the fifth (4.6 ± 1.9) pulse following that echo. In contrast, we estimated that the echo direction possibly affected the direction of the third (3.0 ± 1.6) pulse during the last flight.

## Discussion

The aim of this study is to use a new approach based on a combination of behavioral experiments and acoustic simulation in order to investigate “echo space” and ultimately gain more knowledge about how bats might perceive their environment acoustically. We tested the hypothesis that as spatial learning progresses, bats pay more attention to the minimum parameters necessary for flight and fly more efficiently, i.e., the location of their attention changes by observing the differences in “echo space” before and after spatial learning. In the experiment, after the bats learned the space, the distribution of echo incidence points was concentrated near the edges of obstacles in the space. This result suggests that bats consider the information from the edge of the obstacle as important spatial information obtained from echolocation behavior when avoiding obstacles. We next examined the hypothesis that bats, which use echolocation to understand space, have some relationship between echo information and flight. Specifically, since there was a correlation between the pulse direction and the turn rate [15], we tested whether there was also a correlation between the echo direction and the turn rate. The results showed that there was a correlation between the direction of the echo and the turn rate and that the peak of the correlation coefficient changed with time before and after spatial learning. Therefore, in order to understand the behavior of bats, including spatial learning while flying an obstacle course, it is necessary to consider not only the pulse direction but also the location of the echo incident points at the same time.

**Fig. 5:**
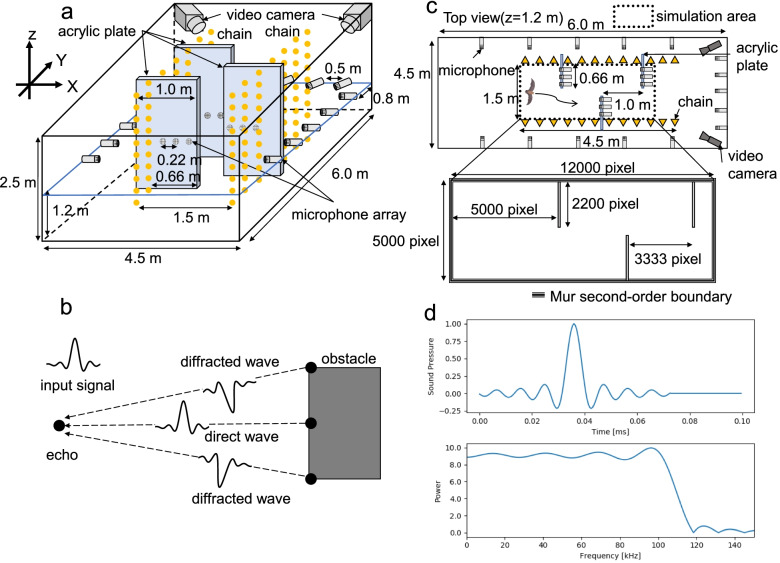
Methods overview. **a** Experimental setup used for the behavioral experiment. The scheme shows the obstacle course setup in which bats were allowed to fly repeatedly. A microphone array was used to measure the pulses of the bats, while a video camera was used to measure their flight trajectories. **b** Scheme of reflected echoes from an object with edges. When a transmitted pulse is directed straight at an object with edges, the echo consists of a direct wave from a point on the object and diffracted waves from the edges of the object. **c** Environment for acoustic simulation (FDTD). A two-dimensional space in the *x*–*y* plane at *z* = 1.2 m in the corridor separated by the chain of the experimental system of the behavioral experiment was used as the space to be analyzed in the simulation. **d** Pulse for acoustic simulation (FDTD). A sinc function waveform with a flat frequency range up to approximately 110 kHz was used in the simulation as the impulse

It had been shown from the examination of pulse direction that bats gaze at the edge of the net when they recognize space [19]. In the present study, the acoustic analysis using the FDTD method revealed that the echo space in which the bats appear to focus was more on the edge after spatial learning. In the case of object identification using images from cameras, many algorithms can be used for edge extraction, such as the Scale-Invariant Feature Transform [20]. In contrast, in sound object recognition, diffracted waves are reflected from the edges as echoes in addition to a direct wave (Fig. 5b and see Additional file 2: Movie S2 and Additional file 3: Movie S3). In other words, in sound-based object recognition, solely information from the direct wave and diffracted wave from the edge is contained in the echoes [21]. In this study, the direct wave from the surface and the diffracted wave from the edge of the acrylic sheet were returned as echoes, and the echo localization point appeared on both sides. It is possible to localize the edge acoustically because the echoes are returned from the edge itself. In contrast to object recognition by images, therefore, sound-based methods may be more efficient because only minimal information is required.

We calculated the peak times of the correlation coefficients between echo direction and turn rate in order to examine the time between the input of the echo to the bat and the change in flight before and after learning. The bats were affected not only by the pulse direction but also the echo direction, and the time of influence was earlier in the echo direction than in the pulse direction. This is a natural result, considering that bats change the characteristics of the following pulse based on information of the previous echoes. For instance, previous studies have suggested that the Doppler shift compensation behavior is based on the echoes of the previous one to three pulses [22], and in the bat’s target direction anticipation behavior, the model best matches the measured data when the target velocity is estimated from the echoes of the previous five pulses [23]. In the present study and during the first flight, we found a positive correlation peak of the pulse direction that preceded the turn rate change by 186 ms, which is similar to the time lag obtained in an obstacle environment in a previous study [5]. In contrast, the echo direction change preceded the turn rate change by 377 ms (Fig. 4a), which suggests that the correlation of the pulse direction with the turn rate is a secondary result of the correlation between the echo direction and the turn rate. Based on our results, we estimated that the echo direction possibly affects the direction of approximately the fifth pulse during the first flight, which is consistent with the results of the above-mentioned studies [22, 23]. In comparison to the first flight, we found that the echo direction possibly affects the direction of earlier pulses during the last flight. Thus, bats might be able to reduce the time required to determine the turn rates and pulse directions after the arrival of echoes when they have become familiar with their surroundings.

Bats estimate the distance to an object based on the time delay between the emitted pulse and the echoes [24, 25]. For vertical object localization, species with tragus use notch information [26], while species with antitragus respond by moving both ears [27]. It is also possible to use the frequency information contained in the echoes to exclude clutter [28]. For information about the horizontal angle of the object, binaural cues for sound localization provide the information [26, 29–31]. In the present study, we calculated the echo incidence points by an engineering method [32] from interaural time difference in the acoustical simulation because by considering the spatial resolution of bats in the horizontal direction [29], the object localization is comparable to that of this method. However, this may be different from the way bats localize objects and it is necessary to reproduce the actual process in bats more precisely in the future. For instance, we should include the directivity of the ear position by introducing the head-related transfer function of the bat, including its ears, in the acoustic simulation space and a moving source to reflect the effect of the Doppler shift during flight to obtain more detailed spatial information. In addition, a bat auditory processing algorithm should be introduced into the post-echo processing such as the spectrogram correlation and transformation model [33–35]. We believe that this approach of echo simulation is a useful first step toward elucidating the perception space by bats. In addition, in the field of sensing engineering, the bat’s sonar has been proposed as a model for object localization [36], obstacle avoidance algorithms [37, 38], and is practically applied to an autonomous robot [32, 39, 40]. Sound-based sensing has been widely utilized to perceive our environment in a simple manner, as it is relatively inexpensive to produce and handles a much smaller amount of data compared to methods based on vision. Further understanding of the sound-based spatial sensing strategies of bats will advance the technology in more unique and new directions.

## Conclusions

In this study, we used a combination of behavioral experiments and acoustic simulations to engineer how bats can detect space from echoes. By visualizing the spatiotemporal changes in the “echo space,” we found that the bats’ perceptual echo space was sharpened by spatial learning to enable efficient navigation and that the bat’s decisions about flight paths in obstacle space are based on the spatial information provided by the preceding echoes. Our research results using bats as a model will be an important step toward elucidating animal decision-making that controls navigation behavior.

## Methods

### Study species

Seven adult Japanese greater horseshoe bats (*Rhinolophus ferrumequinum nippon*, three males and four females) were used for the behavioral experiment. The bats of this species emit pulses consisting of a short initial FM component, a constant frequency component (CF), and a terminal FM component. These pulses are accompanied by overtones. Among the overtones, the second overtone, which has a CF component of approximately 68–70 kHz, has the highest sound pressure [11].

### Experimental setup and procedure

The experiment was conducted in a corridor [4.5 m (L) × 1.5 m (W)] bordered by chains within a flight chamber [9 m (L) × 4.5 m (W) × 2.5 m (H)]. Three acrylic panels [1 m (W) × 2 m (H)] were placed in the corridor next to the chain-walls, alternating on the left and right sides, with each panel being spaced 1 m apart from each other (Fig. 5a). The seven bats were allowed to fly one after the other from the starting position through the obstacle course to a net behind the starting position 12 times. At the time of their first flight, they were completely unfamiliar with the obstacle course. In addition, the experimenter carried each bat to the starting position of the flight while covering the bat with his hands to prevent it from collecting information about its surroundings. After a bat had flown through the course once, the experimenter immediately captured the bat using a 50 × 50-cm insect net. The bat was then allowed to drink water from a plastic pipette and was brought back to the starting point by the experimenter who was covering them with their hands while carrying. During the experiment, only infrared lights were used in the flight chamber. Two high-speed video cameras (MotionPro X3; IDT Japan, Inc., Tokyo, Japan; 125 frames per second) recorded the flight paths of the bats, and microphones arranged in an array around the flight chamber recorded the emitted pulses of the bats to calculate the direction of pulse emission. We also calculated the pulse emission timing of the bats by measuring the pulses they emitted using a telemetry microphone attached to the bat’s head. Please refer to the publication of Yamada et al. [14] for further methodological details.

### Finite-difference time-domain (FDTD) method

We used the two-dimensional FDTD method to simulate the echoes that return to the positions of the right and left ears of bats during their flight. The FDTD method is one of the numerical methods developed by Yee to solve Maxwell’s equations, which are the governing equations of electromagnetic waves [41]. It has been widely used in the field of acoustics since Madariaga adapted it for elastic waves [42]. The method uses the governing equations of motion and the continuity of sound pressure as given in Eqs. (1) and (2).

1 $$\frac{{\boldsymbol\partial}{\boldsymbol p}} {{\boldsymbol\partial}{\boldsymbol t}}+{\boldsymbol\rho}{{\boldsymbol c}_{\mathbf0}}^{\mathbf2}{\boldsymbol\nabla}\cdot{\boldsymbol u}=\mathbf0$$

2 $${\displaystyle \begin{array}{c}\frac{\boldsymbol{\partial u}}{\boldsymbol{\partial t}}+\frac{\mathbf{1}}{\boldsymbol{\rho}}\mathbf{\nabla}\boldsymbol{p}=\mathbf{0}\end{array}}$$

where *p* is the sound pressure, *u* is the particle velocity vector, ***ρ*** is the medium density, and ***c***_**0**_ is the speed of sound. The following equations are obtained by discretizing the above governing equations on a staggered grid.

3 $${\displaystyle \begin{array}{c}{\boldsymbol{p}}_{\boldsymbol{i},\boldsymbol{j}}^{\boldsymbol{n}+\mathbf{1}}={\boldsymbol{p}}_{\boldsymbol{i},\boldsymbol{j}}^{\boldsymbol{n}}-\frac{\boldsymbol{\rho} {{\boldsymbol{c}}_{\mathbf{0}}}^{\mathbf{2}}\Delta \boldsymbol{t}}{\Delta }\left({\boldsymbol{u}}_{{\boldsymbol{x}}_{\boldsymbol{i}+\frac{\mathbf{1}}{\mathbf{2}},\boldsymbol{j}}}^{\boldsymbol{n}+\frac{\mathbf{1}}{\mathbf{2}}}-{\boldsymbol{u}}_{{\boldsymbol{x}}_{\boldsymbol{i}-\frac{\mathbf{1}}{\mathbf{2}},\boldsymbol{j}}}^{\boldsymbol{n}+\frac{\mathbf{1}}{\mathbf{2}}}+{\boldsymbol{u}}_{{\boldsymbol{y}}_{\boldsymbol{i},\boldsymbol{j}+\frac{\mathbf{1}}{\mathbf{2}}}}^{\boldsymbol{n}+\frac{\mathbf{1}}{\mathbf{2}}}-{\boldsymbol{u}}_{{\boldsymbol{y}}_{\boldsymbol{i},\boldsymbol{j}-\frac{\mathbf{1}}{\mathbf{2}}}}^{\boldsymbol{n}+\frac{\mathbf{1}}{\mathbf{2}}}\right)\end{array}}$$

4 $${\displaystyle \begin{array}{c}{\boldsymbol{u}}_{{\boldsymbol{x}}_{\boldsymbol{i}+\frac{\mathbf{1}}{\mathbf{2}},\boldsymbol{j}}}^{\boldsymbol{n}+\frac{\mathbf{1}}{\mathbf{2}}}={\boldsymbol{u}}_{{\boldsymbol{x}}_{\boldsymbol{i}+\frac{\mathbf{1}}{\mathbf{2}},\boldsymbol{j}}}^{\boldsymbol{n}-\frac{\mathbf{1}}{\mathbf{2}}}-\frac{\Delta \boldsymbol{t}}{\boldsymbol{\rho} \Delta }\left({\boldsymbol{p}}_{\boldsymbol{i}+\mathbf{1},\boldsymbol{j}}^{\boldsymbol{n}}-{\boldsymbol{p}}_{\boldsymbol{i},\boldsymbol{j}}^{\boldsymbol{n}}\right)\end{array}}$$

5 $${\displaystyle \begin{array}{c}{\boldsymbol{u}}_{{\boldsymbol{y}}_{\boldsymbol{i}+\frac{\mathbf{1}}{\mathbf{2}},\boldsymbol{j}}}^{\boldsymbol{n}+\frac{\mathbf{1}}{\mathbf{2}}}={\boldsymbol{u}}_{{\boldsymbol{y}}_{\boldsymbol{i}+\frac{\mathbf{1}}{\mathbf{2}},\boldsymbol{j}}}^{\boldsymbol{n}-\frac{\mathbf{1}}{\mathbf{2}}}-\frac{\Delta \boldsymbol{t}}{\boldsymbol{\rho} \Delta }\left({\boldsymbol{p}}_{\boldsymbol{i}+\mathbf{1},\boldsymbol{j}}^{\boldsymbol{n}}-{\boldsymbol{p}}_{\boldsymbol{i},\boldsymbol{j}}^{\boldsymbol{n}}\right)\end{array}}$$

where ***∆*** is the grid interval and ***∆t*** is the time resolution. The terms ${\boldsymbol{p}}_{\boldsymbol{i},\boldsymbol{j}}^{\boldsymbol{n}}$, ${\boldsymbol{u}}_{{\boldsymbol{x}}_{\boldsymbol{i},\boldsymbol{j}}}^{\boldsymbol{n}},$ and ${\boldsymbol{u}}_{{\boldsymbol{y}}_{\boldsymbol{i},\boldsymbol{j}}}^{\boldsymbol{n}}$ are the sound pressure and particle velocity at a position (x, y) = (i***∆***, j***∆***) and time t = n***∆t***, respectively. The sound propagation is calculated by alternately solving Eqs. (3), (4), and (5). In addition, because FDTD is a time-domain and not a frequency-domain acoustic simulation, diffuse attenuation is included, while absorption attenuation is not.

### Visualization of echo incidence points

#### Simulation space setup

In the behavioral experiment, the obstacle walls (acrylic plates) were placed from the floor to the ceiling, so that the bats avoided them by changing their horizontal flight path. In other words, vertical (*z*-direction) flight had almost no effect on the avoidance behavior of this experimental system, and in fact, little change in altitude was observed during flight. Therefore, considering the amount of computation, we created a two-dimensional space (*x*-*y*) at the height level of array microphones (*z* = 1.2 m) to reproduce this setting in the simulation space. The FDTD simulation space was located inside the corridor (4.5 m × 1.5 m) as shown in Fig. 5c, and the absorption boundary condition was the Mur second-order boundary condition [43]. As presented in Additional file 4: Table S1, the Courant–Friendrichs–Lewy (CFL) number was 0.57. The spatial resolution (*dx*) was 0.3 mm. Moreover, the densities of air and acrylic were 1.29 and 1.18 kg/m^3^, with a bulk modulus of 142.0 × 10^3^ and 8.79 × 10^9^ Pa, respectively.

#### Echo simulation

To simulate the echoes that returned to the position of the bats’ ears, we used information on the bats’ pulse emission position and direction obtained from the behavioral experiment. A sinc function signal with a wide frequency band and high time resolution was used in the simulation space as the emitted pulse. It was flat up to 110 kHz and the duration was 0.073 ms (Fig. 5d). The sinc function signal was emitted from two source positions (simulating the bat’s nostrils), which were set at 1.25 mm each on the left and right side from a given pulse emission position and in the direction of emission which was both obtained from the behavioral experiment (Additional file 5: Fig. S1a; note that 1.25 mm is based on the half wavelength of the CF2 frequency of *R. ferrumequinum nippon* (68 kHz)) [44, 45]. The directivity of the sound source is created by two point sources that are close to each other. Receiver positions (simulating the bat’s ears) of the returning echoes in the simulation were set at 10 mm left and right from the pulse emission points (note that 10 mm is based on the distance between the ears of *R. ferrumequinum nippon*) (Additional file 5: Fig. S1a). In the simulation, the impulse responses (echoes) at the two receiver positions were calculated using the FDTD method. Then, the bat’s pulse was convolved with these impulse responses to obtain echoes. The bat’s pulses in the simulation were the downward FM components created up to the third harmonic, which has been reported to be used for distance discrimination [46]. The downward FM component has a duration of 2 ms and the first harmonic decreases linearly from 34 to 25 kHz, corresponding to the terminal FM component of the echolocation pulse in *R. ferrumequinum nippon* (Additional file 5: Fig. S1b). The first and third harmonics were set to a sound pressure level of -40 dB based on the second harmonic [47]. The sampling frequency was 2 MHz (1/dt). When pulses are emitted from the two point sources, they propagate in a forward and backward direction (Additional files 1, 2, and 3: Movies S1, S2, and S3). Therefore, in order to exclude echoes from the back side of the bat, the simulation was performed by excluding obstacles located behind the source positions.

#### Calculation of echo incidence points

Bats estimate the distance to an object based on the time delay between the emitted pulse and the echoes [24, 25]. In the simulation, this echo delay was calculated by cross-correlating the simulated left and right echoes with the bat’s pulses. The peak value of the autocorrelation of the pulse was normalized, and the peak time above the threshold of the cross-correlating signal was extracted for the left and right echoes (Additional file 6: Fig. S2). The threshold value was set to 0.02 to detect almost all the peaks. The left and right peak times were matched within a window of the maximum time difference between the two receiving points (2.5 mm/340 m/s = 7.4 μs), and combinations of the left and right peak times ([*t*_*r*_, *t*_*l*_]) were obtained. The echo incidence points ((*x*_echo_, *y*_echo_)) were then calculated by solving the following two elliptic equations determined by the calculated peak times using the pulse emission positions obtained in the behavioral experiments (i.e., the centers of the two source positions in the simulation) and the left and right receiver positions in the simulation space as the focal points. The pulse emission position was set to *y* = 0 and the direction of pulse emission was set to *x* = 0.

6 $${\displaystyle \begin{array}{c}\frac{{\left({\boldsymbol{x}}_{\boldsymbol{echo}}-{\boldsymbol{x}}_{\boldsymbol{r}\mathbf{0}}\right)}^{\mathbf{2}}}{{{\boldsymbol{a}}_{\boldsymbol{r}}}^{\mathbf{2}}}+\frac{{\left({\boldsymbol{y}}_{\boldsymbol{echo}}-{\boldsymbol{y}}_{\boldsymbol{r}\mathbf{0}}\right)}^{\mathbf{2}}}{{{\boldsymbol{b}}_{\boldsymbol{r}}}^{\mathbf{2}}}=\mathbf{1}\end{array}}$$

7 $$\begin{array}{l}\frac{\left({\boldsymbol x}_{\boldsymbol{echo}}-{\boldsymbol x}_{\boldsymbol l\mathbf0}\right)^{\mathbf2}}{{\boldsymbol a}_{\boldsymbol l}^{\mathbf2}}+\frac{\left({\boldsymbol y}_{\boldsymbol{echo}}-{\boldsymbol y}_{\boldsymbol l\mathbf0}\right)^{\mathbf2}}{{\boldsymbol b}_{\boldsymbol l}^{\mathbf2}}=\mathbf1\\{\boldsymbol a}_{\boldsymbol r}=\frac{{\boldsymbol t}_{\boldsymbol r}\cdot{\boldsymbol c}_{\mathbf0}}{\mathbf2},{\boldsymbol b}_{\boldsymbol r}=\sqrt{{\boldsymbol a}_{\boldsymbol r}^{\mathbf2}-\left({\boldsymbol x}_{\boldsymbol r}-{\boldsymbol x}_{\boldsymbol r\mathbf0}\right)^{\mathbf2}}\\{\boldsymbol a}_{\boldsymbol l}=\frac{{\boldsymbol t}_{\boldsymbol l}\cdot{\boldsymbol c}_{\mathbf0}}{\mathbf2},{\boldsymbol b}_{\boldsymbol l}=\sqrt{{\boldsymbol a}_{\boldsymbol l}^{\mathbf2}-\left({\boldsymbol x}_{\boldsymbol l}-{\boldsymbol x}_{\boldsymbol l\mathbf0}\right)^{\mathbf2}}\end{array}$$

where *x*_*r*0_ is the center position between the pulse emission position and the right receiver position, and *x*_*l*0_ is the center position between the pulse emission position and the left receiver position (see Additional file 7: Fig. S3). An animation of the process of echo incidence point visualization using acoustic simulation is shown in the Additional file 3: Movie S3.

### Statistical analysis

#### Effect of spatial learning on the echo incidence point distribution

We were interested in testing whether the positions of echo incidence points on the obstacle walls changed depending on the spatial learning status of the bats (first vs. last flight). Since the bat’s task in the behavioral experiment was to avoid the inner sides of the obstacle walls, we considered those echo incidence points that were located on the inner half of the walls (*n* = 1058) and calculated the distance of these points to the inner edge. We modeled this data as a function of the bat’s spatial learning status (first vs. last flight) using generalized linear mixed effect models (function glmmTMB, package glmmTMB_1.0.2.1) [48] assuming a negative binomial error distribution (nbinom1) due to overdispersion. Because several echo incidence points can result from one pulse and several pulses were used per bat, we included a random effect with a pulse-ID nested within the bat-ID. The quality of the model fit was graphically examined (function in package DHARMa_0.3.3.0) [49] and its overall significance was determined by comparing it to the respective null model that contains only the random effect via a *χ*^2^-test (function anova in stats) [50]. The significance of the factor coding for the bat’s experience was derived from a type-II-Wald- *χ*^2^-test (function anova, package car_3.0-10) [51] while the factor-levels were compared based on least-square-means (function lsmeans, package emmeans_1.5.4) [52]. The statistical significance level was set at *p* = 0.05.

#### Effects of spatial learning on flight path planning

We calculated the turn rate at 1 ms intervals from the acquired flight paths. The turn rate is the time derivative of the flight path. To investigate the relationship between the turn rate and the pulse and echo direction, respectively, we shifted the turn rate data by a time lag of *τ* in 10 ms steps from − 100 ms (to the left) up to + 600 ms (to the right) relative to the pulse and echo direction, respectively, and calculated the corresponding correlation coefficients. The 95% confidence intervals for the correlation coefficients were determined by a Fisher transformation of the correlation coefficients to the following ranges:

8 $${\displaystyle \begin{array}{c}\pm {\boldsymbol{z}}_{\boldsymbol{\alpha} /\mathbf{2}}\frac{\mathbf{1}}{\sqrt{\boldsymbol{n}-\mathbf{3}}}\end{array}}$$

where n represents the number of data and ***z***_***α***/**2**_ is 1.96 for 95% confidence interval.

Then, we extracted the *τ*-values that were associated with the highest correlation coefficients for each bat and each category (first vs. last flight; *n* = 28) and modeled them on the scale of seconds as a function of the degree of spatial learning (first vs. last flight) in interaction with the factor describing the type of information used by the bat (echo vs. pulse) using linear mixed effect models (function lmer, package lme4_1.1-26) [53]. We added the bat-ID as a random effect to the model due to the repeated sampling of the same individuals. The quality of the model fit, the significance of factors within the model, and the comparisons between factor-levels were conducted using the same functions as mentioned above. The overall model significance was tested against the respective null model using parametric bootstrapping (function PBmodcomp, package pbkrtest_0.5-1.0) [54].

## Supplementary Information

**Additional file 1: Movie S1.** The reflection wave of sound when it is incident at an angle to a circular object. Two point sources are used to add directivity to the sound source, and the pulse direction and echo direction are displayed assuming that the flight direction is 0 degrees (to the right of the video). The red arrows indicate pulse direction, and the green arrows indicate echo direction. No diffraction wave is generated, and the direct wave returns as an echo (note that the pulse direction and the echo direction are the same).

**Additional file 2: Movie S2.** The reflection wave of sound when it is incident at an angle on a wall. Two point sources are used to add directivity to the sound source, and the pulse direction and echo direction are displayed assuming that the flight direction is 0 degrees (to the right of the video). The red arrows indicate pulse direction, and the green arrows indicate echo direction. The direct wave (main echo) is reflected from the surface and the diffracted echoes are reflected from both edges of the wall. All echo directions do not coincide with the pulse direction.

**Additional file 3: Movie S3.** The movie depicts the flight path as well as the pulse direction and the simulated echo incidence points. The waveforms in this movie are based on Gaussian monopulses, and the surrounding walls are characterized by Mur's first-order equation. The obstacle course area is 2/25 times smaller than the area in the simulation used in this study.

**Additional file 4: Table S1.** Acoustic simulation parameters.

**Additional file 5: Figure S1.** Source and receiver positions for acoustic simulation and the convolutional signal for echo simulation. (a) In the acoustic simulation, the source positions (red circles) were placed at two points 1.25 mm to the left and right from the pulse emission position obtained from the behavioral experiment. The receiver positions (red triangles) were placed at two points 10 mm to the left and right from the pulse emission position, which is the distance between the two ears of the bat. (b) Overview of convolutional signals (FM signal) created for the simulation of echoes at the receiver positions.

**Additional file 6: Figure S2.** Overview of simulated echoes and cross-correlation results. (a) Oscillogram and spectrogram of the pulse used in the simulation. (b) Oscillogram and spectrogram of a simulated echo at the position of the left receiver. (c) Oscillogram and spectrogram of a simulated echo at the position of the right receiver. (d) Cross-correlation results between the pulse and echo at the position of the left receiver. The red circles are the acquired peak positions. (e) Cross-correlation results between the pulse and echo at the position of the right receiver. The red circles are the acquired peak positions.

**Additional file 7: Figure S3.** Conceptual diagram of how echo incidence points were calculated using two ellipses.

## Data Availability

The data used in this study and the code used in the analysis are available at figshare, Teshima, Yu; Yamada, Yasufumi; Tsuchiya, Takao; Heim, Olga; Hiryu, Shizuko (2022): Visualization of bat echo space by using acoustic simulation. figshare. Dataset. (10.6084/m9.figshare.19100633.v1). In addition, we used the following dataset from Yamada et al. [14]. Yamada, Yasufumi; Mibe, Yurina; Yamamoto, Yuya; Ito, Kentaro; Heim, Olga; Hiryu, Shizuko (2022): row dataset for article entitled “Modulation of acoustic navigation behaviour by spatial learning in the echolocating bat Rhinolophus ferrumequinum nippon”. figshare. Dataset. (10.6084/m9.figshare.19102712.v1).
