# Supplementary material for: Analysis of echolocation behavior of bats in “echo space” using acoustic simulation
Source: BMC Biol. 2022 Mar 14;20:59. doi: 10.1186/s12915-022-01253-y (PMC8919609; doi:10.1186/s12915-022-01253-y)
Supplement: Supplementary file 4 — Additional file 4: Table S1. Acoustic simulation parameters. [file 12915_2022_1253_MOESM4_ESM.pdf]

**Courant-Friendrichs-Lewy (CFL) number**		**0.57**
**Space resolution (dx) [mm]**		**0.30**
**Density [kg/m^3]**	**air**	**1.29**
	**acrylic**	**1.18**
**Bulk modulus [Pa]**	**air**	**1.42×10^5**
	**acrylic**	**8.79×10^9**
