# Supplementary figures and images for: Analysis of echolocation behavior of bats in “echo space” using acoustic simulation
Source: BMC Biol. 2022 Mar 14;20:59. doi: 10.1186/s12915-022-01253-y (PMC8919609; doi:10.1186/s12915-022-01253-y)

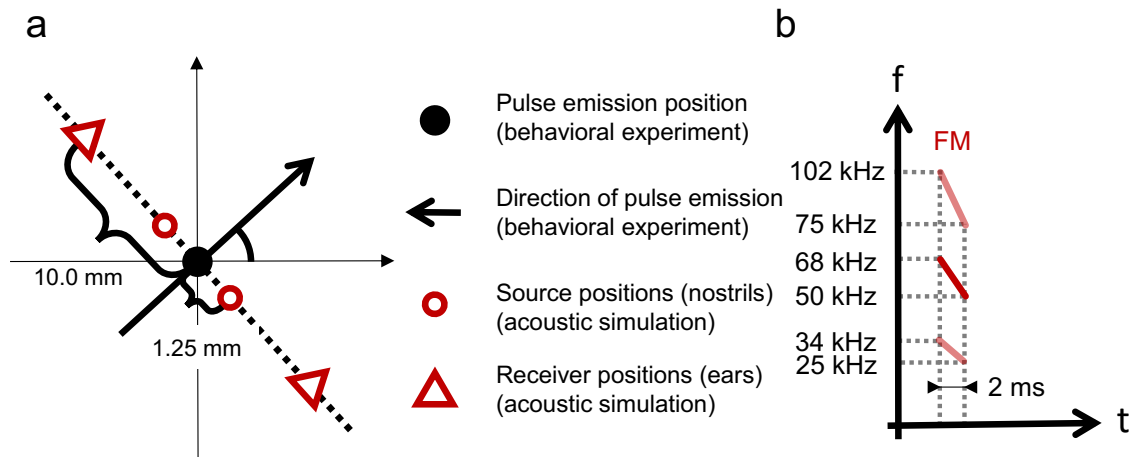

Supplement: Supplementary file 5 — Additional file 5: Figure S1. Source and receiver positions for acoustic simulation and the convolutional signal for echo simulation. (a) In the acoustic simulation, the source positions (red circles) were placed at two points 1.25 mm to the left and right from the pulse emission position obtained from the behavioral experiment. The receiver positions (red triangles) were placed at two points 10 mm to the left and right from the pulse emission position, which is the distance between the two ears of the bat. (b) Overview of convolutional signals (FM signal) created for the simulation of echoes at the receiver positions. [file 12915_2022_1253_MOESM5_ESM.pdf]

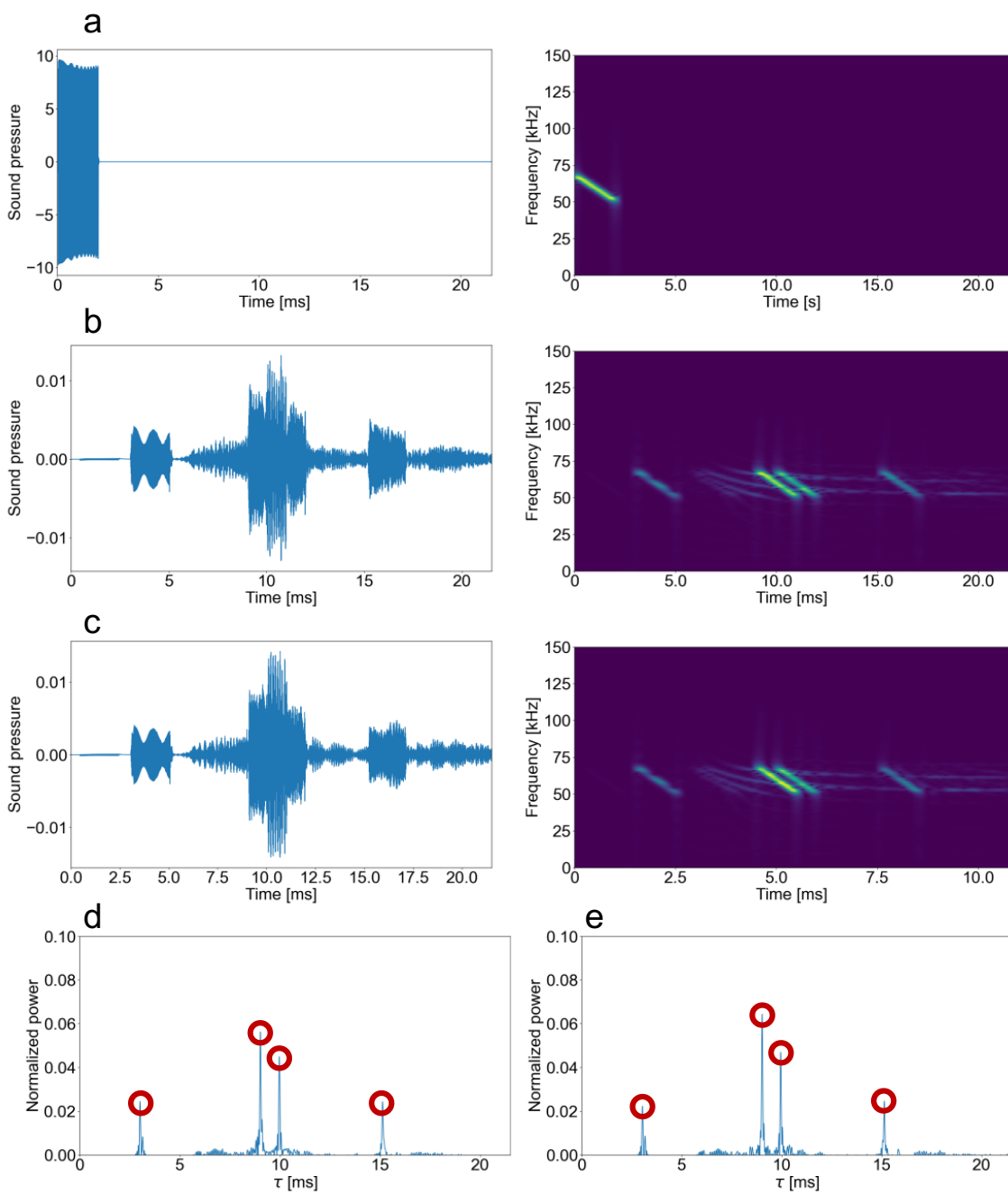

Supplement: Supplementary file 6 — Additional file 6: Figure S2. Overview of simulated echoes and cross-correlation results. (a) Oscillogram and spectrogram of the pulse used in the simulation. (b) Oscillogram and spectrogram of a simulated echo at the position of the left receiver. (c) Oscillogram and spectrogram of a simulated echo at the position of the right receiver. (d) Cross-correlation results between the pulse and echo at the position of the left receiver. The red circles are the acquired peak positions. (e) Cross-correlation results between the pulse and echo at the position of the right receiver. The red circles are the acquired peak positions. [file 12915_2022_1253_MOESM6_ESM.pdf]

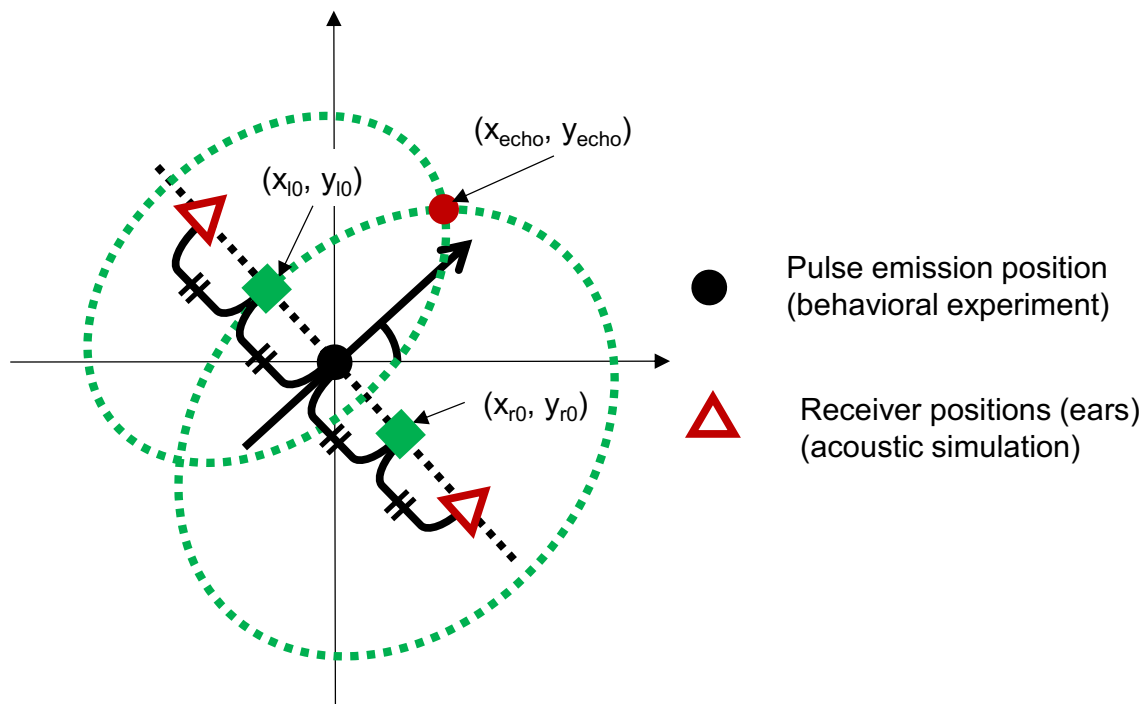

Supplement: Supplementary file 7 — Additional file 7: Figure S3. Conceptual diagram of how echo incidence points were calculated using two ellipses. [file 12915_2022_1253_MOESM7_ESM.pdf]
